# Spatiotemporal analysis of a population management intervention for dogs and cats in a municipality in central Mexico

**DOI:** 10.14202/vetworld.2024.1693-1701

**Published:** 2024-08-04

**Authors:** Miguel Galarde-López, Erika del Rosario Rosales-Moreno, Sandra Elizabeth Hernández-Méndez, Alejandra Rubí Medina-Cháirez, Angélica Denisse Villavicencio-López, Mauricio Pineda-Mundo, Obed Núñez-Ruíz, Antonio Reyna-Sevilla

**Affiliations:** 1 National Center for Disciplinary Research in Animal Health and Safety, National Institute of Forestry, Agricultural and Livestock Research, Mexico City, 05110, Mexico; 2 Tequisquiapan Animal Health and Welfare Services Center, Querétaro, 76775, Mexico; 3 Faculty of Veterinary Medicine and Zootechnics, Autonomous University of Tamaulipas, Tamaulipas, 87274, Mexico; 4 Center for Teaching, Research and Extension in Animal Production in the Plateau, Faculty of Veterinary Medicine and Zootechnics, National Autonomous University of Mexico, Querétaro, 76790, Mexico; 5 School of Public Health of Mexico, National Institute of Public Health, Morelos, 62100, Mexico; 6 Directorate of Medical Benefits, Mexican Institute of Social Security, Mexico City, 06400, Mexico

**Keywords:** cat, dog, overpopulation, public health, spatiotemporal, sterilization program

## Abstract

**Background and Aim:**

The overpopulation of dogs and cats has generated socioeconomic, political, and animal welfare problems, in addition to an important public health problem, due to the risk of zoonotic diseases. This study aimed to analyze the spatiotemporal coverage of canine and feline sterilization services provided by a governmental agency in the rural and urban areas of the municipality of Tequisquiapan, Querétaro.

**Materials and Methods:**

This cross-sectional study was conducted in Tequisquiapan’s municipality, Querétaro, Mexico, from July 2019 to September 2022. The total number of sterilized dogs and cats was obtained from the monthly records of the Tequisquiapan Animal Health and Welfare Services Center (CESSBA, by its Spanish acronym). The collected information was related to the sterilized animals (species and sex) and their responsible guardians (sex and address). Access to dog and cat sterilization services was assessed using a geographic information system. Kernel density and directional ellipse tools were used to analyze the CESSBA coverage of care. Indicators were estimated to compare magnitudes and changes at the census tract level.

**Results:**

A total of 4,489 animals were sterilized, with n = 2,611 (58%) dogs, of which 1,939 were female and 672 were male. The remaining n = 1,878 animals were cats, representing 42% of the total, with 1,257 females and 621 males. Up to 73% of the sterilized animals were owned by women. The population management of dogs and cats allowed us to increase the territorial coverage from 71.8% in 2019 to 92.3% in 2022. According to the temporal analysis (2019–2022), there was an annual upward trend in the number of sterilizations performed by CESSBA, with a rate of between 55.6 and 94.3 registered sterilizations per 100 inhabited dwellings and between 166.4 and 302.8 registered sterilizations per 1000 inhabitants.

**Conclusion:**

The analysis of the dog and cat sterilization service coverage revealed an upward trend, consisting of an increase in accessibility and participation of responsible caregivers who resided in both urban and rural areas of Tequisquiapan. Although it was not possible to evaluate the impact of the program, the use of georeferenced data and geospatial analysis showed that it can support the control of animal overpopulation.

## Introduction

The overpopulation of dogs and cats is a public health problem due to the risk of attacks (bites) and the transmission of zoonotic diseases such as rabies [1]. The World Health Organization (WHO) estimates that more than 60 million rabies cases are associated with dog bites worldwide [2]. Some studies have reported that dog and cat aggressions with zoonotic potential are more frequent in rural areas, mainly due to the presence of free-roaming dogs, as well as the degree of marginalization and the lack of public services for humans and animals [3–5]. In addition, dog and cat overpopulation can also be associated with other socioeconomic, political, and animal welfare problems, such as the risk of accidents on public roads, attacks on livestock, and the loss of endemic wildlife [6]. Due to the interaction, social benefits, and potential risks of zoonoses in the human-animal relationship, the World Organization for Animal Health and the WHO have promoted strategies for managing dog and cat populations as an essential part of the one health initiative [7, 8]. In this regard, reproductive control programs involving sterilization are the most widely used strategies to control the size of the dog and cat population, and depending on the approach taken, these strategies reduce public health risks [9–11].

High densities of free-roaming dogs can be a problem. However, the abundance of dogs and cats varies in different regions and countries, depending on the habitat type (rural/urban) and human population (density and sociocultural factors) [12]. In Mexico, an estimated 43 million dogs and 16 million cats are distributed in approximately 25 million households [13]. However, homeless/sheltered (free-roaming or feral) dogs or cats could account for up to 70% of the population [14, 15]. Although it is estimated that there are 790,000 dogs and 260,000 cats in Queretaro, no specific data are available at the municipal level [13]. Animal population management has been approached in diverse ways for different purposes, including long-term sheltering, culling, public education on responsible ownership, and surgical sterilization of dogs and cats; this has been the most widely used strategy [13, 16] The main groups responsible for establishing dog population management programs have been government agencies, researchers, and/or civil society organizations focused on animal welfare [13, 17]. However, few studies have analyzed the coverage of sterilization programs and access to the service from a geographic perspective, which allows for identifying the territorial extension of the services offered, analyzing patterns and changes in each period, as a tool to support actions on animal population management. Moreover, other studies have used geo-referencing tools to visualize and analyze canine aggressions or the coverage of anti-rabies vaccination programs [18–21].

The appropriate use of geospatial tools can support reformulating strategies and public policy plans aimed at managing dog and cat overpopulations, especially in the field of public health [22, 23].

To the best of our knowledge, no epidemiological research in Mexico has used spatial analysis tools to examine the territorial scope of canine and feline sterilization services. Therefore, this study aimed to analyze the spatiotemporal coverage of canine and feline sterilization services provided by a governmental agency in the rural and urban areas of Tequisquiapan, Querétaro.

## Materials and Methods

### Ethical approval

Ethical approval was not necessary for this study. The use of the database was authorized by the Animal Health and Welfare Services Center (CESSBA) of Tequisquiapan. The confidentiality of the responsible guardians was maintained.

### Study period and location

**Figure-1:**
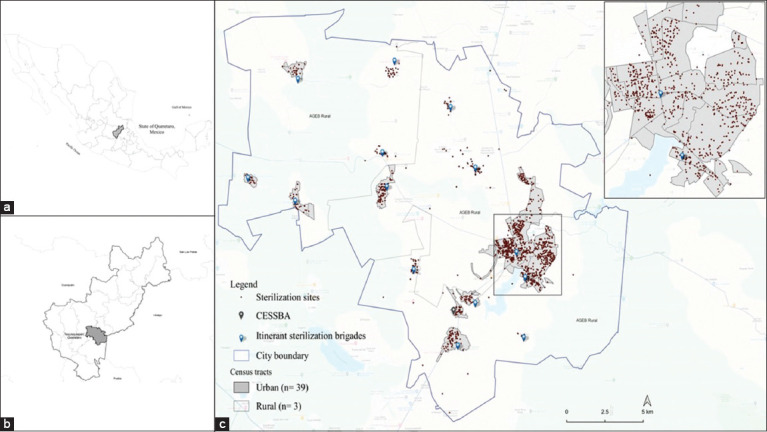
(a-c) Map of census tracts and distribution of sterilized dogs and cats in the municipality of Tequisquiapan, Querétaro, during the period 2019–2022 [Source: The map was prepared by the authors according to the National Geostatistical Framework and records of the Tequisquiapan Animal Health and Welfare Services Center].

This cross-sectional descriptive study was based on the monthly records of the canine and feline sterilization services performed by the Tequisquiapan Animal Health and Welfare Services Center (CESSBA, by its Spanish acronym), corresponding from July 2019 to September 2022. CESSBA is a government agency, located in the municipality of Tequisquiapan, Querétaro. Tequisquiapan is one of the municipalities of the state of Queretaro in central Mexico (Figure-1a), it has an area of 343.6 Km2, and its coordinates are 20°31’14” north and 99°53’45” west, with an average annual temperature of 19°C (Figure-1b). The political-administrative delimitations of the 42 Urban Basic Geostatistical Areas (39 urban and 3 rural), also known as census tracts (including their population), were obtained from the National Geostatistical Framework report based on the most recent Population and Housing Census. The reported population for 2020 was 72,201, comprising 51.5% women and 49.5% men, distributed in 19,000 homes. The urban census tracts comprise 83% of the municipality’s population. Sixty percent of the inhabitants have only basic education [24].

### Data source

To address the health problems and needs of the municipality’s companion animals, CESSBA’s facilities offer preventive medicine, short-term shelter, adoption, culling, cremation, and sterilization services. However, as a strategy to control the animal population and expand access to sterilization services, some itinerant sterilization brigades were implemented in different communities in the rural and urban areas of the municipalities, as well as a “mega sterilization campaign” in the municipal capital (Figure-1c).

### Data collection and spatiotemporal analysis

From the monthly CESSBA records on sterilization services performed during the study period, data were obtained on the number of sterilizations performed according to species and sex. In addition, the sex of the guardian responsible for the animal and her address (geographic coordinates) were obtained to compare the use and access to the sterilization service according to sex and geographical location. Descriptive analysis of the variables of interest was performed using the statistical program Stata version 15 (StataCorp LLC, College Station, Texas, USA) [25]. The initial phase of the georeferencing and geospatial distribution analysis consisted of using the geographic coordinates attributed to the addresses of the guardians and specific data to visualize the geographic distribution (Figure-1c) using Google Maps® (Google LLC, California, USA) [26].

This map was the basis for the spatiotemporal analysis using point data to identify the absolute and relative frequencies of dog and cat sterilization performed at the census unit level (urban and rural). The estimated directional ellipses for each year were used to analyze the spatiotemporal coverage of dog and cat population management [27]. The Kernel density estimation tool was employed to depict a parameter that had been modified to align with the local scale of the study area at the census unit level in accordance with the count of sterilizations conducted per km^2^ [28]. This approach facilitated the observation of regions within municipalities with the highest and lowest accentuated spatial density. The evaluation of the coverage, as well as the maps, was executed using the QGIS software version 3.22.3 (Creative Commons Corporation, Mountain View, California, United States).

### Standardized rate estimation

In the next phase, to analyze the coverage of access to dog and cat sterilization services provided by the CESSBA, the following metrics were calculated at the census unit level: the rate of dog and cat sterilizations per 100 inhabited dwellings and the rate of dog and cat sterilizations per 1000 inhabitants. Based on data from the National Self-Reported Welfare Survey, it is estimated that in the municipality of Tequisquiapan, there are between 27,000 and 40,000 dogs and cats, with approximately 2–3 animals per home [13, 24, 29]. To mitigate the small numbers associated with indicators in tiny census tracts during rate calculations, the empirical Bayes method was used to adjust for disparities in the number of inhabitants reported in each census tract [30, 31]. Furthermore, the values of these indicators were classified into quintiles to identify the lowest and highest outliers in the study area. In addition, indicator values were classified into quintiles to discern the most extreme values within the study region, both at the lower and upper ends.

## Results

During the study period, 4,489 sterilizations were recorded. Of these, 58% were dogs (1,939 females and 672 males), and 42% were cats (1,257 females and 621 males). Seventy-three percentages (3,275) of the sterilized animals had a responsible guardian. In addition, 52.4% of the sterilized animals were female (dog/cat) with female-responsible guardians, compared with 8.3% of sterilized males (dog/cat) with male-responsible guardians (Table-1).

**Table-1:** Distribution of sterilized dogs and cats, by sex of the responsible guardians.

Sex responsible guardian	Animal	Sex	n = 4,489 (%)
Women n = 3,275	Dog n = 1,809	Female	1,360 (30.2)
		Male	449 (10.0)
	Cat n = 1,466	Female	993 (22.1)
		Male	473 (10.5)
Men n = 1,214	Dog n = 802	Female	579 (12.8)
		Male	223 (4.9)
	Cat n = 412	Female	264 (5.8)
		Male	148 (3.3)

Source: Prepared by the authors according to records of the Tequisquiapan Animal Health and Welfare Services Center

The highest quantity of sterilization was spatially referenced in the southeastern region of the township and in the urban census tracts (Figure-1c). The map of the municipality of Tequisquiapan generated with the distribution of sterilized dogs and cats by census tracts showed that 81.5% (n = 3,656) of the sterilized animals were georeferenced in 37/39 urban census tracts, representing 99 sterilizations (M = 71, SD = 83.5) per census tract. Meanwhile, 18.5% (n = 833) of the sterilized animals were georeferenced in the three rural census tracts, representing 277 sterilizations per census tract (Figure-1c). The distribution of sterilization performed in the three rural census tracts was as follows: southeast zone 39.0% (323/883), central zone 36.9% (306/883), and northwest zone 24.0% (199/883).

Two urban census tracts recorded the highest number of sterilizations, with 19.1% (n = 697/3,656) of dogs and cats sterilized by the CESSBA. Both census units were located in the southeast of the municipality. Furthermore, two of the 39 urban census tracts did not generate any records of sterilized dogs or cats.

**Figure-2:**
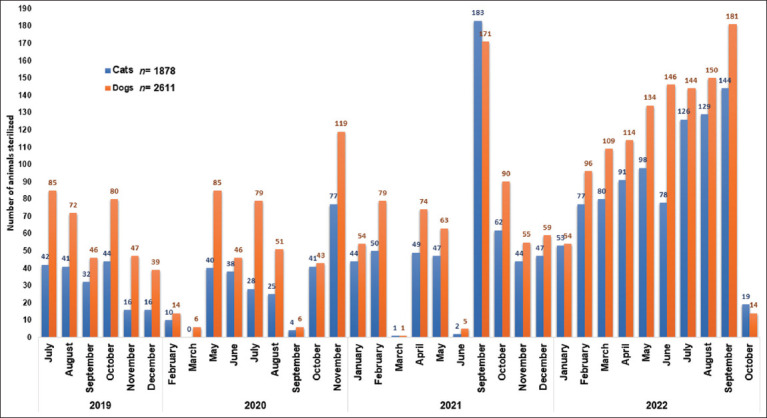
Temporary trend of dog and cat sterilizations performed in the municipality of Tequisquiapan, Querétaro, during 2019–2022 [Source: The map was prepared by the authors according to records of the Tequisquiapan Animal Health and Welfare Services Center (CESSBA)].

According to the temporal analysis (2019–2022), there was an annual increase in the number of sterilizations performed by CESSBA. In 2019, there were 560 (12.5%) sterilizations; in 2020, there were 712 (15.9%); in 2021, there were 1180 (26.3%); and by 2022, there were 2,037 (45.4%). In September 2021, due to a “mega campaign” to sterilize dogs and cats, a spike in the number of sterilizations carried out was recorded. By 2022, an increase in the number of sterilizations performed was due to the incorporation of veterinary students who collaborated as social service providers at CESSBA. The frequency of dog and cat sterilization performed during the study period is shown in Figure-2.

**Figure-3:**
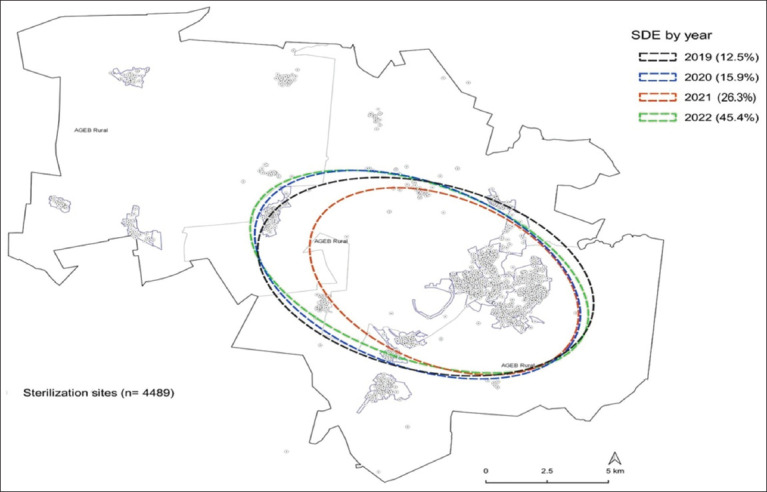
Temporary coverage of the canine and feline sterilization service in the municipality of Tequisquiapan, Querétaro, during 2019–2022 [Source: The map was prepared by the authors according to the National Geostatistical Framework and records of the Tequisquiapan Animal Health and Welfare Services Center].

**Figure-4:**
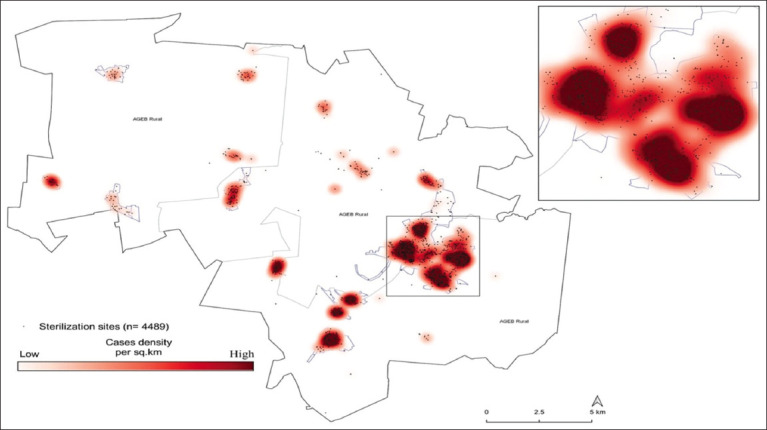
Spatial density of the canine and feline sterilization service in the municipality of Tequisquiapan, Querétaro, during 2019–2022 [Source: The map was prepared by the authors according to the National Geostatistical Framework and records of the Tequisquiapan Animal Health and Welfare Services Center (CESSBA)].

**Figure-5:**
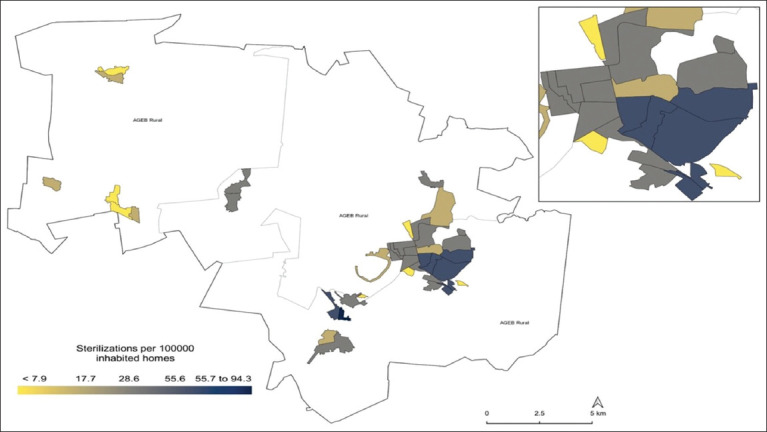
Sterilization coverage by 100 inhabited dwellings in Tequisquiapan, Querétaro, during 2019–2022 [Source: The map was prepared by the authors according to the National Geostatistical Framework and records of the Tequisquiapan Animal Health and Welfare Services Center].

**Figure-6:**
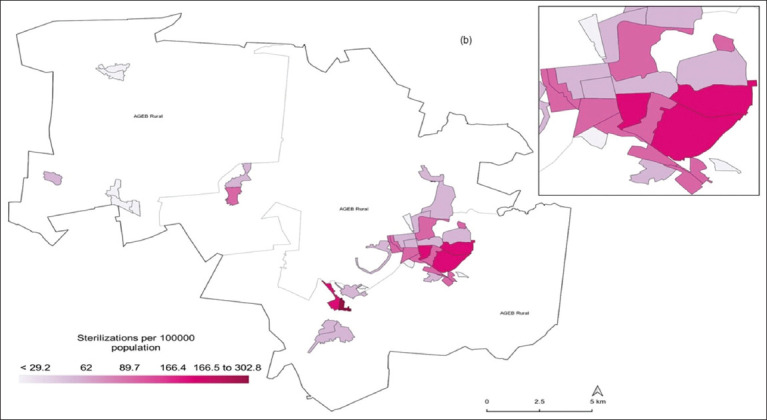
Sterilization coverage by 1000 inhabitants in Tequisquiapan, Querétaro, during 2019–2022 [Source: The map was prepared by the authors according to the National Geostatistical Framework and records of the Tequisquiapan Animal Health and Welfare Services Center].

The directional ellipse tool showed that 27/39 (69.2%) urban census tracts were located within the study area’s extent and in a direction that coincided with the urban population patch. This included 87.7% (3,207/4,489) of the dog and cat sterilization performed during the study period (Figure-3). At the census tract level, the central and southwestern areas of the municipality had a density of 0.3–1.4 sterilizations of dogs and cats per square kilometer, while in the northern and northwestern areas, the density was <0.2 sterilizations per square kilometer (Figure-4). Based on the sterilization coverage of dogs and cats carried out by CESSBA, a rate of between 55.6 and 94.3 registered sterilizations per 100 inhabited dwellings was estimated (Figure-5), and a rate of 166.4–302.8 registered sterilizations per 1,000 inhabitants (Figure-6).

## Discussion

This study demonstrated that the territorial coverage of the canine and feline sterilization services provided by a government agency (CESSBA) has gradually increased each year. The urban population has greater access to and use of the service than the rural area of Tequisquiapan, Querétaro. At present, there is only one estimated census of dogs and cats in the State of Querétaro; however, it can be estimated that in the municipality of Tequisquiapan, there are between 27,000 and 40,000 dogs and cats [13]. From these data, one can infer that approximately 10% of the canine or feline population within the municipality has undergone sterilization procedures performed by the CESSBA.

In our research, we observed a sterilized dog: cat ratio of 1.3:1, despite the discrepancy with figures documented in state-level animal population censuses that indicated a dog: cat ratio of 3:1. The above shows the use and availability of dog and cat sterilization services by the guardians responsible for the animals. Furthermore, the proportion of dogs and cats sterilized by CESSBA differs from the results of a study conducted in Brazil, in which researchers affirm that cat guardians are less responsible for the sterilization of their animals [11]. A study conducted in Namibia reported that dogs are sterilized more frequently than cats and found no differences associated with the sex of the responsible guardians [32].

A sterilized female: male ratio of 2.4:1 was also observed. This preference for sterilizing females more frequently than males could be associated with different factors; however, it is consistent with a previous study by Rojas *et al*. [33], which reported that offspring are produced by females and not by males, so females are more likely to be sterilized. For example, a study conducted in Yucatan, Mexico, found more sterilized female dogs than male dogs [34]. Some other studies conducted in Peru and the United States agree that female dogs and cats are more likely to be sterilized than male dogs and cats [33, 35]. This tendency might be a factor that affects the efficiency of sterilization services because some dogs/cats ecology studies have reported that there are usually more males than females in an animal population [20, 36].

In our study, we observed that a higher percentage of women opted for sterilization services for their pets. Nevertheless, Barni *et al*. [11] reported that characteristics such as the sex of the responsible guardian did not influence responsible pet ownership. However, sociocultural norms, as well as individual attitudes leading to pet sterilization decisions, are factors to be considered in future studies [32, 37, 38]. A study conducted in New Zealand revealed that men were less likely to sterilize their pets than women, arguing that they were concerned about the “sexual integrity” of the animal, which could arise from equating the “sexuality or masculinity” of the animals to that of their responsible guardians [39]. On the other hand, Glasser *et al*. [40] reported that men were more likely than women to think that sterilization could influence the “behavior” of animals.

In countries such as Mexico, the problem of canine and feline overpopulation has been addressed through different actions, including reproductive surgical management, animal welfare legislation, education on responsible guardians, and animal registration [8, 41, 42]. Although these initiatives are designed to effectively manage animal populations in a local context, population management plans should also cover indicators related to animal populations, human-animal interactions, the environment, and public services offered to the population [43].

It was estimated that 70% (13,300/19,000) of households in Tequisquiapan keep an average of 2–3 pets, while 75% (54,000/72,000) of residents own a minimum of one pet. Our findings showed that a range of 0.5–1 animal per household or 0.1–0.3 animals per individual underwent sterilization [13]. It is important to recognize that 83% of people reside in urban census tracts, which could explain the concentration of sterilization services for dogs and cats in these areas [24]. This phenomenon has been observed in several places, although it is not necessarily comparable [10, 36, 44, 45]. This highlights the need to expand the coverage of dog and cat sterilization services to rural areas to reduce potential gaps in their use.

Regarding the socioeconomic levels of responsible dog and cat guardians, some studies have mentioned that human populations with better economic and educational levels, such as those in urban areas, have better access to veterinary services. This may be because the companion animal population is subject to the same social and health determinants as the human population, resulting in a direct or indirect impact of the same risk factors [43].

Although the CESSBAA dog and cat sterilization services expanded their territorial presence from 71.8% to 92.3% (2019–2022) in the census units, the coverage pattern did not show changes in the territory. The northwest of the municipality has not been covered, so sterilization services could reach total territorial coverage once the omitted areas are identified, added to the use of the services by the guardians to multiply the number of sterilized animals. Research on animal population dynamics modeling in Iran has revealed that 50% annual sterilization coverage in the female dog population will result in a 44% reduction in the free-roaming dog population compared with the baseline population [20]. However, this study proposes only female sterilization coverage models as an effective method for dog population control. In this sense, the results of our research can serve as evidence to adjust possible changes in the coverage of sterilization services in different areas from a geographical perspective, combined with the number of dogs and cats present in each census tract.

Mexican states and municipalities have implemented plans and strategies to regulate the population growth of dogs and cats. With sterilization being one of the main ways to control dog and cat populations. Still, there is a lack of adequate legislation and a surveillance system that provides information and evaluate the effectiveness of these actions as part of comprehensive management with the effective allocation of resources in priority areas with the support of geospatial tools [43]. In this regard, the roaming strategy for medical equipment implemented by CESSBA considered the characteristics of the local context, the needs of the population, and the public resources available to improve access to and use of dog and cat sterilization services.

Our study has some limitations due to the type of epidemiological design, lack of a local animal census, limited data in medical records, or the implementation of other actions as part of a dog and cat control program. However, this was a turning point in estimating the use of sterilization services by a government agency in rural and urban areas by analyzing geospatial data.

## Conclusion

The analysis of the dog and cat sterilization service coverage revealed a consistent trend in accessibility and use among accountable caretakers residing in both urban and rural areas of Tequisquiapan. An adequate analysis of the coverage of dog and cat sterilization services is essential to allocate human, financial, and material resources to help control the animal population, safeguard public health, mitigate animal welfare problems, and achieve the implementation of strategies.

## Authors’ Contributions

MGL, ERRM, ARS: Study design, data extraction, statistical analysis, and drafted the manuscript. MGL and ARS: Conceived and designed the study and drafted and revised the manuscript. SEHM, ARMC, ADVL, MPM, and ONR: Carried out the study. SEHM, ARMC, ADVL, MPM, and ONR: Supervised the study. All authors have read, reviewed, and approved the final manuscript.
